# Pre-Clinical Tools for Predicting Drug Efficacy in Treatment of Tuberculosis

**DOI:** 10.3390/microorganisms10030514

**Published:** 2022-02-26

**Authors:** Hasmik Margaryan, Dimitrios D. Evangelopoulos, Leticia Muraro Wildner, Timothy D. McHugh

**Affiliations:** 1 UCL Centre for Clinical Microbiology, Division of Infection & Immunity, UCL, Royal Free Campus, London NW3 2PF, UK; l.wildner@ucl.ac.uk (L.M.W.); t.mchugh@ucl.ac.uk (T.D.M.); 2 Department of Microbial Diseases, Eastman Dental Institute, UCL, Royal Free Campus, Rowland Hill Street, London NW3 2PF, UK; d.evangelopoulos@ucl.ac.uk

**Keywords:** tuberculosis, drug activity, in vitro preclinical modelling, MDR-TB, synergism, transcriptomics, high order combinations, drug combinations, drug efficacy

## Abstract

Combination therapy has, to some extent, been successful in limiting the emergence of drug-resistant tuberculosis. Drug combinations achieve this advantage by simultaneously acting on different targets and metabolic pathways. Additionally, drug combination therapies are shown to shorten the duration of therapy for tuberculosis. As new drugs are being developed, to overcome the challenge of finding new and effective drug combinations, systems biology commonly uses approaches that analyse mycobacterial cellular processes. These approaches identify the regulatory networks, metabolic pathways, and signaling programs associated with *M. tuberculosis* infection and survival. Different preclinical models that assess anti-tuberculosis drug activity are available, but the combination of models that is most predictive of clinical treatment efficacy remains unclear. In this structured literature review, we appraise the options to accelerate the TB drug development pipeline through the evaluation of preclinical testing assays of drug combinations.

## 1. Introduction 

Tuberculosis (TB, Table S2: Abbreviation) is caused by the human pathogen *Mycobacterium tuberculosis*, which claimed the lives of 1.5 million people in 2020 (WHO REPORT 2021 [1]). The World Health Organisation (WHO) reported a large global drop in the number of patients newly diagnosed with TB from 7.1 million to 5.8 million in 2019 and 2020, respectively, mainly due to the COVID-19 pandemic. The total number of people with multidrug-resistant (MDR) and drug-resistant (DR)-TB enrolled on treatment from 2018 to 2020 was 482,683, only 32% of the 5 year target (2018–2022) of 1.5 million [1,2]. Poor treatment completion rates in MDR-TB are often the consequence of the requirement for treatment for a longer duration with second line drugs, which are less effective and have greater toxicity than the four drugs (isoniazid (INH), rifampicin (RIF), ethambutol (EMB), and pyrazinamide (PZA)) most commonly used to treat drug-susceptible TB (DS-TB) [3,4]. 

Treatment failure is associated with *M. tuberculosis* strains that are initially DS, acquiring resistance or through infection with already antibiotic-resistant bacteria [3,4]. Multitude factors contribute to the difficulty of successfully treating MDR-TB. These include the diversity of clinical disease presentation, varied drug penetration into pathological lesions, in vivo bacterial phenotypes, intrinsic drug resistance and the continued survival of drug-tolerant and persisting populations, as well as a limited number of validated drug targets and the requirement for combination drug therapy [5,6,7].

In an attempt to meet this complex challenge, the approach to designing new MDR-TB regimens was changed in 2016, taking into account the effectiveness and safety of standardised shorter regimens (lasting up to 12 months) and the effect of surgical interventions on treatment outcomes for drug-resistant TB. Whilst there was considerable progress in exploring shortened treatment regimens (6–9 months or less) in phase II/III trials using new and existing antituberculosis medications in novel combinations (Nix-TB trial, STREAM TB study, SimpliciTB, TB-PRACTECAL) [8,9,10,11], these were not preceded by a systematic evaluation of the clinical significance of in vitro drug interactions on efficacy. In vitro, pharmacological data suggested that the use of new drugs such as Bedaquiline (BDQ), Pretomanid (Pa) and Linezolid (LZD) has the potential to improve DR-TB treatment outcomes, and indeed subsequent clinical trials have confirmed this. Despite this, in clinical practice, the use of LZD in combination with XDR-TB treatment showed favourable outcomes [12,13]; it is important to monitor adverse reactions when using LZD in the long term TB treatment. Additionally, depending on the mechanism of RIF resistance when combined with LZD, careful attention is required to avoid the development of resistant mutants [14].

There is scope for improving the evidence base prior to committing to Phase II or III studies. BDQ was recommended by the WHO in June 2013 under specific conditions [15], and subsequently, from 2018, it was widely used in MDR-TB treatment regimens, and updates were incorporated into the WHO consolidated guidelines in 2020 [4]. The successful results of the NiX-TB trial, which led to the registration of Pa by the USA Food and Drug Authority (FDA) and European Medicines Agency (EMA) for the treatment of XDR, treatment-intolerant or non-responsive MDR pulmonary TB [16], provide hope for the ambitious target of a pan-TB regimen that is effective, short (2 months), and is active against both DS- and MDR-TB.

In this structured literature review, we summarise transcriptomic and in vitro drug interaction studies for *M. tuberculosis* which may inform the development of new regimens. All available evidence on the approach and interpretation of *M. tuberculosis* drug interaction results was pooled to create a framework for the evaluation of anti-TB drug combinations as a potential regimen. We aim to identify how synergistic or antagonist drug interactions affect the efficacy of combination therapies and which high throughput methods were used to address the question.

## 2. Methods

### Study Selection and Search Strategy

This structured literature review was conducted in accordance with the Preferred Reporting Items for Systematic Reviews and Meta-Analyses (PRISMA) statement [17].

To retrieve relevant articles, a systematic electronic database search was performed in Medline via PubMed, Google Scholar, Scopus, Web of Science, the Central Register of Controlled Trials (CENTRAL), in the Cochrane Library, and EBSCO libraries, WHO International Clinical Trials Registry Platform and complemented by a search of bibliographies of relevant articles. The literature search was restricted to studies published in English from January 2011 to May 2020 that reflected increased activity in the evaluation of new drugs to treat MDR-TB and XDR-TB in short-course regimens, and in consequence, new efficacy data became available. An electronic form was created to curate the data from the selected studies.

The search terms used were combinations of the keywords: “transcriptomics”, ‘’Multidrug-Resistant Tuberculosis”, “MDR-TB”, “Extensively Drug-Resistant Tuberculosis”, “XDR-TB”, Fractional inhibitory concentration (’’FIC”), Minimum Inhibitory Concentration (‘’MIC‘), synergetic’’, drug–drug interaction (‘’DDI’’), ‘’antagonistic’’, ‘’SCR’, ‘’Factorization’’, ‘’BDQ’’, ‘’Delamanid (DLD)’’, ‘’LZD’’, ‘’Clofazimine (CFZ)’’, and ’’Moxifloxacin (MFX)’’. In addition, the references of the chosen articles and relevant review papers were hand-searched and reviewed.

Duplicate articles retrieved from PubMed and Google Scholar were removed. If the standard search returned numerous results which were not relevant, then the search was refined.

The search results were assessed to find original study publications evaluating drug combinations, changes of treatment efficacy (synergistic or antagonistic drug interactions) with the use of combined diagnostic tools such as the drug susceptibility test (DST), MIC, and or genotyping methods.

**Figure 1:**
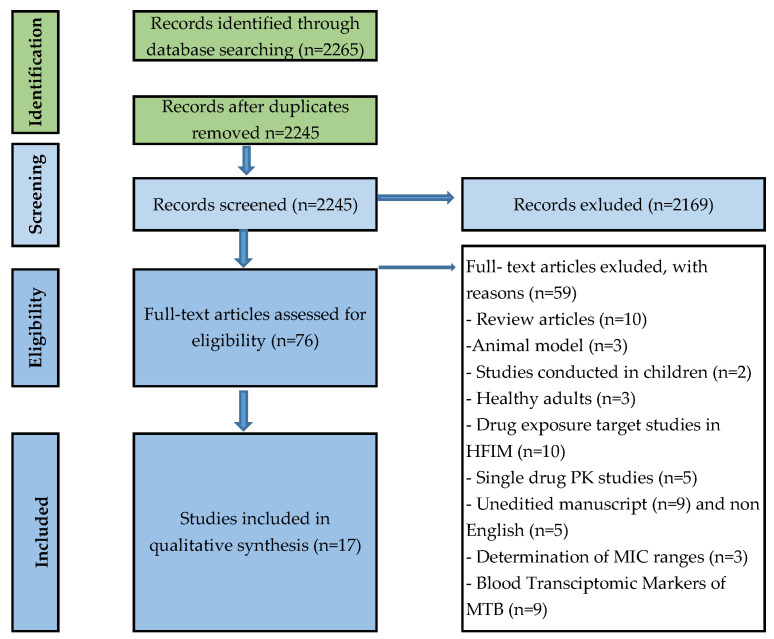
PRISMA flowchart outlining the number of each publication at each stage of the structured literature review.

First, the titles and abstracts were checked for eligibility. When studies were classified as eligible, a copy of the entire article was downloaded to apply inclusion criteria (Figure 1).

There are no validated tools for risk of bias assessment concerning drug synergy or antagonism studies. In the absence of such a tool, the risk of bias in the study was assessed by noting the presence or absence of essential components required for adequate interpretation of results of a drug interaction. This provided the opportunity to narratively compare the included studies on the risk of bias related to the methods and design.

The following components were checked: total sample size, the inclusion of drug-susceptible and drug-resistant clinical isolates and or reference strains (e.g., H37Rv), drug daily dose if applicable, description of specimen handling, use of validated analytical methods such as MIC, the FICI to evaluate the combination effect, early bactericidal activity (EBA) to assess the potency of new anti-tuberculosis drugs in clinical studies, and area under the concentration–time curve (AUC) calculation, to determine whether a study had a high, medium, or low risk of bias. Studies were considered low risk of bias when ≥5 culture isolates were used. The risk of bias assessment of the included studies is provided in Table 1.

## 3. Results

As our focus was studies that evaluated how synergistic or antagonist drug interactions affect the efficacy of combination therapies in *M. tuberculosis* and which efficient measurement methods were used for the analysis and interpretation of drug interactions, the studies were found to be mostly retrospective or confirmatory in nature and lacked a comparison group. 

Studies that reported complete information on drug efficacy, synergism, and antagonism with culture-confirmed DS, MDR-TB, and/or XDR-TB cases using clinical and or laboratory samples in in vitro experiments were selected.

The literature search yielded 2665 reports; 2589 were excluded during the initial title and abstract screening as not relevant. After a full article review of 76 studies, 17 studies were eligible for inclusion as summarised in the Preferred Reporting Items for Systematic Reviews and Meta-Analyses (PRISMA) flowchart provided in Figure 1. Three studies (17.6%) reported transcriptomics signatures with the use of high throughput methods, and 14 studies (82%) concerned in vitro models for the confirmation of synergistic or antagonistic drug interaction in clinical and laboratory samples. The small number of transcriptomic studies might be because the methods used have become more accessible in recent years, and the understanding of pharmacokinetics and the mechanism of action of new anti-TB drugs is still developing. Although studies did address transcriptomic signatures during treatment, no prospective clinical trial was found that was designed to address the use of transcriptomic signatures to predict the drivers of drug synergy and clinical regimen efficacy in *M. tuberculosis***.**

### 3.1. In Vitro Microbiological Based Assays Using In Vitro Checkerboard Models

The accurate prediction of clinically relevant antibiotic synergy based on in vitro testing was always the goal of TB clinical trials. In order to develop quantitative and reproductive assays for antimicrobial activity estimation when two or more drugs are combined, the synergism of the drugs is studied using checkerboard assay (solid medium and micro-both dilution assays), detecting the MIC of anti-tuberculosis drugs in combination schemes [18,20,21,22,23,29,31,32]. The combinatorial effects in these studies are determined by the measurement of the fractional inhibitory concentration index (FICI), which describes the interaction between two antimicrobials [35,36]. In all studies reported in this review, the results were interpreted as synergism (FICI < 0.5), no interaction (FICI > 0.5 but < 4)), and antagonism (FICI > 4).

Ma et el. used in vitro checkerboard assays and the high-throughput diagonal measurement of n-way drug interaction (DiaMOND) method to validate predictions from the inferring drug interactions using the Chemo-Genomics and Orthology (INDIGO-MTB) computational model (Table 2) and Table S1 (Summary of the drug targets, products and mechanism of action). The INH-RIF-STR combination was found to be synergistic [33], while pairwise combinations of INH-STR and INH-RIF were identified as antagonistic. A comparison of the in vitro checkerboard data with transcriptomic data produced under in vitro broth culture conditions and in vivo drug interaction was obtained.

Strong agreement was reported between in vitro synergy and in vivo sputum culture negativity; however, combinations involving BDQ and CFZ alone or in a three-drug combination with PZA, ethambutol (EMB), RIF, or INH were all found to be synergistic with poor clinical outcomes. The RIF-MFX combination was identified to be antagonistic in the INDIGO MTB model and in the in vitro checkerboard assay but had good in vivo efficacy [38]. Despite being antagonistic, it suppresses the evolution of resistance, and therefore synergy alone does not always suggest clinical efficacy.

The REDCA results in drug interaction studies were promising, reporting variation of mean FIC values in comparison to classical checkerboard (FICI equal to 1 for REDCA and 0.75 for classical checkerboard)**.** However, the current microtitre plate systems containing a 9-by-8 matrix of concentration do not allow for the evaluation of the effect of the drugs on the suppression of resistance. This is because the probability of a resistant colony developing is very low due to the small bacterial load in each well [18]. Although the study conducted by Zhang et al. [39] found synergy in only 33.3% and 20.8% of MDR strains against CFZ/MFX and CFZ/CAP combinations, respectively, Li et al. reported synergy in 21 (70.00%) *M. tuberculosis* strains against the CFZ/CAP combination and 29 (96.67%) against the CFZ/MFX combination when the minimum FICIs were calculated. This could be explained by the use of the checkerboard method and a difference in concentration range to that reported by Zhang et al.

Miranda-Silva et al. [22] used a low-complexity in vitro system (Greco URSA model) where *M. tuberculosis* strains are presented with fixed concentrations of antibiotics. The data indicated that Pa and MFX are a promising combination for the killing of NRP *M. tuberculosis*, showing a favourable outcome towards bacteria in the log and acid phases. It is considered that they would have a lower potency against the metabolic state having the lowest growth rate in NRP [22]. A similar study conducted by Miranda-Silva and colleagues attempted to characterise LZD and BDQ interaction in both metabolic states (log and acid phases). They reported that the LZD and BDQ interaction is additive for bacterial killing for both metabolic states.

### 3.2. In Vitro Time-Kill Kinetic Assay

In vitro time kill-kinetics assays can be adopted in a predictive preclinical modelling framework to assess anti-tuberculosis drug activity. This provides important information on the mycobacterial killing dynamics alone and in combination as well as to the selection of drug resistance. Due to this, the time kill-kinetics assay detects differences in anti-TB drug activities that would not have been identified with the use of classical drug susceptibility assays, such as the MIC determining only mycobacterial growth inhibition [18,28,29]. Bax et al. determined the role of in vitro time-kill kinetics assays [28]. The concentration- and time-dependent mycobacterial killing ratio of STR, INH, RIF, EMB, PAS, and PZA drugs was determined by single drugs or in dual, triple, and quadruple combination exposure against the *M. tuberculosis* Beijing genotype to assess drug synergy and the prevention of resistance emergence. In vitro ranking based on the strength and rate of mycobacterial killing showed that STR, RIF, and INH were the most powerful anti-TB drugs. STR and INH showed the most rapid bactericidal activity, while STR and RIF showed a sterilising function against fast-growing, extracellular *M. tuberculosis*. The INH/RIF combination showed synergistic activity at clinically used concentrations, and none of the other dual, triple, or quadruple drug combinations achieved synergy in this model. Both RIF and STR prevented the selection of INH resistant mutants at clinically used concentrations.

PAS and PZA showed little activity due to the low pH of the medium (6.6 to 5.6) that compromised mycobacterial growth. This impacted the inhibition of mycobacterial growth in the unexposed control sample. It could also be argued that PZA activity against this mycobacterial subpopulation is not desirable as PZA lacks activity against fast-growing *M. tuberculosis*, which is in accordance with its early bactericidal activity (EBA) data [40].

Although the time-kill method is a reliable predictor of in vivo synergy, the disadvantage of this method is that it depends on the effect of inoculum size and there are difficulties in the interpretation of results; the percentage of dead cells calculated relative to the growth control by determining the colony-forming unit (CFU/mL) or living cells of each tube using the agar plate count method [20,28,29].

### 3.3. In Vitro Models: Use of the Hollow Fibre Infection Model

The in vitro hollow fibre infection model (HFIM), a continuous flow culture system that allows the dynamic manipulation of culture conditions [41], allows pharmacological modelling of drug–drug interactions and shows that anti-TB drug effectiveness is often better reflected by the AUC divided by the MIC ratio [19,30]. This model allows the concentration–time profiles observed in patients to be mimicked for single drugs and with combinations by evaluating exposure measures for the ability to kill *M. tuberculosis* under different physiologic conditions and drug regimens [30,41].

Drusano et al. [19] evaluated LZD and RIF combinations in the HFIM using a fully parametric mathematical model to study the behaviour of the regimen for a population of patients. The impact of LZD and RIF alone and in combination against the log phase *M. tuberculosis* H37Rv was tested using URSA. LZD and RIF interaction was additive but with an insignificant predisposition to act antagonistically to killing the wild-type (WT) population.

The study showed a major change in RIF MIC with a 32-fold increase with the LZD combination. As LZD is acting alone on these organisms and suboptimal exposures simply lead to the amplification of the resistant population. Additionally, the failure to suppress resistance indicated that this drug combination would not achieve a shortened duration of therapy with standard doses of RIF.

Louie et al. used the HFIM to study the MFX regimen in the different phases of *M. tuberculosis* growth and evaluated growth quantitatively by the culture of bacterial suspensions from the HFIM [30]. The data produced provides a comparison with the MFX plus Pa combination that was incorporated into the TB-PRACTECAL regimen BDQ/Pa/LZD/MFX, where there was good activity demonstrated against MDR TB (ClinicalTrials.gov identifier NCT02589782).

### 3.4. Theoretical/Mathematical Models Used to Identify Potential Regimens

Peterson et al. used INDIGO MTB with the Environment and Gene Regulatory Influence Network (EGRIN) [33,34,42] and Probabilistic Regulation of Metabolism (PROM) computational models and studied synergistic mechanisms of BDQ-tolerance regulons [34]. BDQ activates a regulatory network that coordinates various resistance mechanisms that push *M. tuberculosis* into a tolerant state where it resists BDQ killing. As a consequence, BDQ kills *M. tuberculosis* relatively slowly (96 h) in comparison to INH and RIF [34]. Slow killing is considered to be indicative of its tolerant state [34], and this information would not be available from MIC data alone.

### 3.5. High-Throughput Combinatorial Screening

Studies conducted by Cokol et al., where a high-throughput combinatorial approach was used, identified synergistic or antagonistic high-order drug combinations against *M. tuberculosis*. This was to initiate a geometric framework to rationally factorise high-order drug interactions into lower-order components using lower-order interaction measurements [31,43]. A pantothenate and leucine auxotrophic strain of H37Rv was used for these experiments. These strains demonstrate similar in vitro and intra-macrophage replication rates, responses to anti-TB agents and whole-genome sequence conservation.

A structured experimental sampling and scoring method, DiaMOND, was used to measure combinations for a number of drugs together with a generalised Loewe additivity model for high-order drug interactions. The model discovered that BDQ + CFZ + INH (one three-way combination) had a strong three-way synergy. The three-way and four-way combinations BDQ + CFZ + RIF and BDQ + Pa + RIF and BDQ + CFZ + INH + RIF and CFZ + INH + Pa + RIF, respectively, were reported as synergistic. The three-way synergy of BDQ + CFZ + INH was validated using a conventional three-dimensional checkerboard assay.

Alternatively, to design high order antibiotic combinations, Yilancioglu and Cokol measured 190 pairwise interactions among 20 antibiotics against *M. tuberculosis* growth using a ranking and exclusion design (R/ED) framework model. The pairwise drug interactions were measured using the diagonal method [31], and all possible high-order combinations were ranked by their strength of synergy and antagonism. To improve the standard three-drug combination with the addition of new drugs and to find four-drug combinations against drug-resistant *M. tuberculosis* populations, Yilancioglu and Cokol modelled a procedure interchanging two-order combinations as a cycling treatment. Cycling of Pa with ethionamide (ETH) and BDQ+ CFZ was the best two-order combination cycling against RIF-resistant *M. tuberculosis*. It was also not appropriate to include BDQ for INH-resistant *M. tuberculosis*, as both drugs have similar actions. BDQ was replaced with fusidic acid (FUS), and cycling of Pa + VAN and FUS  +  CFZ was found to be the best two-order combination. For MDR strains, CFZ  +  FUS and lassomycin (LAS) + Pa cycling were the best options. It was found that the cycling of CFZ  +  INH and ETO  +  RIF is considerably superior to the four-order combination in predicting static and lytic synergy scores.

## 4. Discussion

Recent years have seen the introduction of new drugs to the anti-tuberculosis drug pipeline, and together with a number of repurposed drugs, these are currently in or entering clinical trials. These studies and the history of TB drug regimen design show that there is treatment-shortening potential, not least the recent TBTC study 31/ACTG A5349 phase three clinical trial (ClinicalTrials.gov NCT02410772) demonstrating a shortened regimen using rifapentine. The challenge remains to determine which of these compounds to prioritise in designing more effective combinations. Testing each novel drug in combination with new and existing anti-tuberculosis compounds using conventional methodologies is a discouraging process.

This review summarises the approaches to determining synergistic and antagonistic drug combinations used for DS/MDR/XDR TB treatment and how this information can inform the selection of combination therapies. Theoretical/mathematical models are used to identify potential regimens of TB treatment with the use of three or more drugs that have high efficacy at low doses and account for the resistance mechanisms of each drug [33,34,42]. Drug discovery studies involve tremendous efforts for the selection and translation of in vitro data into in vivo animal models to evaluate the efficacy of the drug. Such models must take into account the evidence that during *M. tuberculosis* infection, a population of bacteria exist in different metabolic states [22,23,30,44,45] thus, a standardised framework is required to assess the relationship between these subpopulations where the bacteria can switch between and drug effect data in vivo bridging exposure from a population pharmacokinetic model. The presence of such phenotypically resistant bacteria within the host could increase the need for extended drug therapy against active and latent tuberculosis infection [44,45].

The use of a computational model such as INDIGO-MTB in the context of the transcriptional regulatory network (TRN) showed that drug synergy and antagonism occur due to coordinated, system-level molecular changes that involve multiple cellular processes [34]; however, the INDIGO algorithm is imperceptive to *M. tuberculosis* molecular responses to drugs in the host conditions. This limitation can be addressed considering the use of *M. tuberculosis* transcriptome profile data in a macrophage environment [34,46], where bacteria have the capacity to survive within the environment of the macrophage. 

New drug combinations can be predicted by the use of regulatory network models (EGRIN and PROM combined models) [33] that introduce possibilities to represent gene states and gene–transcription factor interactions. This allows the differentiation of the effect of a drug as compared to non-specific *M.tuberculosis* stress responses. EGRIN and PROM combined models, together with INDIGO, showed a great promise in forming the selection of drug regimens to carry forward to an evaluation in clinical trials. Direct targets were not identified for many of the compounds; thus, metabolic, kinetic, and statistical modelling has limited power in this context. Empirical approaches based on drug similarity or dissimilarity are less effective in predicting interaction outcomes for new drug classes, and they also lack a model for antagonism [47,48,49].

Greco et al. used a URSA modelling strategy [22] as a pre-screen incorporating PK data to demonstrate the bacterial elimination achieved by drug combinations in a therapeutic regimen. Further expansion of this model is required to analyse susceptible and resistant populations and regimens that ideally have synergistic or at least additive effects on bacterial killing and or suppression of resistance prior to the next step: evaluation in an in vitro HFIM [19,30,50].

Discontinuous dosing, especially when the bacterial load is high, has a particular risk for the emergence of drug resistance [18,22,23]. Therefore, the use of mathematical models that evaluate combination regimens or cell elimination and suppression of resistance along with HFIM allows the rational choice of a combination regimen.

Many studies tested log-phase organisms and did not test acid environments and non-replicative persister forms and or intracellular persisters. Most studies used only the *M. tuberculosis* laboratory reference strain H_37_Rv [19,22,23,30,31,32], but it is well established that clinical isolates vary in their phenotype with respect to drug responses and a range of well-characterised clinical isolates should be tested [19,22,23,30].

The findings from studies with laboratory strains do not show the full picture; therefore, we could benefit from the use of validated clinical strains. Gagneux et al. and colleagues suggested broadening the scope of basic and translational TB research by incorporating a set of genetically well-characterised clinical strains typical of the known phylogenetic diversity of the pathogen [51].

Although the checkerboard assay is a useful first step to assess the efficacy in clinical development phases and to test new antituberculous drug regimens, it does not have a standardised methodology for interpretation of assays that include more than two drugs thus leading to reporting of divergent results [35,52]. A systematic approach must be taken in the definition of the FICI cut off, and this needs to be validated. In the interpretation of synergy data, it is important to take into account the clinical usefulness and the statistical principle of FICI, as in drug combination studies, antibiotics are considered as synergistic if the MIC of each drug is 0.25 times or less of the MIC of each drug used alone [36].

An area that is underdeveloped is the nature of the host environment; it is important to note that drug penetration into macrophages and, ultimately, tubercular lesions will have a significant impact on drug efficacy. The time-kill kinetics in vitro assays will provide important information regarding the bactericidal activity of anti-TB drugs during the early phase of treatment, but there is a need for an integrated modelling framework to account for the complexity of the host. Importantly, the in vitro checkerboard technique may predict synergy/antagonism which is not observed in vivo. Time-kill and checkerboard results can be diametrically contrasted when synergy is compared with antagonism, and so results obtained from one model may not be automatically interchangeable with another model [18,20,21,22,23,24,25,26,27,29,31,32,33]. It should also be noted that variables such as drug concentrations, clinically achievable concentrations, fractions of the MIC, and bacterial inoculum size can affect the definitions of synergy [20,28,29].

The main outcome parameters in clinical trial studies are the degree of culture conversion after two months of intensive treatment and the prevention of disease relapse; these two parameters reflect the sterilizing capacity of anti-TB drug regimens [53]. An example of this is the REMox trial [38], which shortened the duration of therapy to 4 months in DS TB patients by switching INH or EMB for MFX in intensive and continuation phases. The RIF and MFX combination showed sputum culture rapid conversion in the first 8 weeks, but this was not sustained in the continuation phase of therapy. It is proposed that the clinical outcomes observed are related to the killing of bacteria in the log phase, while the results in the continuation phase are associated with the slow killing of persister organisms. These results were supported by an in vitro HFIM study demonstrating an antagonistic interaction between these drugs killing NRP organisms [30]. To evaluate the in vivo activities of the most active compounds, a murine model is widely used; however, these were excluded from this review due to the lack of quantitative raw data and variation in metrics. As it was shown in the study conducted by Lopez-Gavin et al., the in vitro results could not be translated to in vivo studies due to variability between animal and human models [21].

## 5. Conclusions

Most of the studies in this review showed that drug combinations are effective against DS/MDR/XDR clinical isolates. However, there is still a need for a clear understanding of the performance of tuberculosis treatment regimens in pre and early phase clinical trials. This is critical in the design of phase III trials and to achieve this standardisation of preclinical models is essential. The use of transcriptomic analysis for understanding drug mode of action provides useful insights into the target pathways affected by drug action. From the data included in this review, we propose that the following must be considered for clinical trial design:

- Studies including three or more drug combinations should test the drug concentration range in separate and combined assays.
- Testing should be performed on bacteria in different metabolic states.
- The use of in vitro methods such as the checkerboard assay is a useful first step; however, a standardised method of interpretation must be validated in all laboratories involved in the studies.
- Drug concentrations used should be pharmacologically relevant.
- Standardised approaches are needed in evaluating all drug combinations in an in vitro HFIM, where drug exposures and human pharmacokinetic profiles of the drug in the target site are simulated to evaluate the impact of these combinations for cell killing and the suppression of resistance [41].

## Figures and Tables

**Table 1:** Risk of bias assessment of the included studies.

First Author, Year	Source of Patients Data	Total Number of Samples Used	TB/DST/MIC Test Results	Material	MIC Value	Validated Analytical Determination/Methodology	Drug Interaction	Sample Handling Described	Endpoints Method AUC Calculation	Endpoints Method FICI Calculation	Endpoints Method EBA Calculation Cmax	Grading Risk of Bias (High, Medium, Low)
Maltempe 2017 [18]	(14 susceptible, 9 INH mono-resistant and 14 MDR and laboratory strains (H37Rv)	37	+, +, +	Culture	RIF (0.004 to 0.25 μg/mL and 4–250 μg/mL). LZD (0.125 to 0.5 μg/mL for susceptible and 0.125–2.5 μg/mL for RIF)	Checkerboard, REDCA assay. Time-kill curve assay	LZD and RIF	+	−	+	−	Low
Drusano 2014 [19]	H37Rv	Not specified	+, −, +,	Culture	LZD (1.0 mg/L) RIF (0.25 mg/L)	HFIM	LZD and RIF	+	−	−	−	High
Calefi- Ferracioli 2013 [20]	H37Rv, 9 susceptible and 10 resistant clinical isolates	19	+, + ^1^, +	Culture	INH, EMB and LFX (0.03–32 mg/L, 0.5–032 mg/L and 0.06–4 mg/L).	REDCA, classical checkerboard assay	INH/ LFX EMB	+	−	+	−	Low
Lopez-Gavin 2015 [21]	7 MDR and 11 DS clinical isolates	17	+, +, +	Culture	CFX, LFX, MFX and UB-8902 (0.0625–1 mg/L); Pa (0.0313–1 mg/L)	Checkerboard	CFZ/Pa/LFX CFZ/Pa/MFX CFX/Pa/Ub-8902	+	−	+	−	Low
Miranda Silva, 2019 [22]	*M. tuberculosis* 18b, H37Rv	Not specified	+, +, +	Culture	MFX (0.25 mg/L and 0.5 mg/L). Pa (0.125 mg/L)	Checkerboard, URSA	MFX and Pa Log, acid, NRP phases	+	−	−	−	High
Miranda Silva, 2018 [23]	*M. tuberculosis* 18b, H37Rv	Not specified	+, +, +	Culture	LZD (1 mg/L) BDQ (0.25–0.5 mg/L), 0.5)	Checkerboard, URSA	LZD and BDQ	+	−	−	−	High
Pang, 2019 [24]	XDR-TB	191 ^2^	+, +, +	Culture	BDQ ≥ 0.063 mg/L, MFXx and GFX (0.125 mg/L), LZD (0.5 mg/L), Cfz (0.25 mg/L)	Checkerboard	BDQ/MFX/GFX/ CFZ, LZD	+	−	+	−	Low
Santos, 2018 [25]	*M. tuberculosis* H37Rv, 2 susceptible and 10 resistant clinical isolate	12	+, +, +	Culture	INH (0.03–6.25 μg/mL) RIF (0.008–100 μg/mL), LFX (0.12–0.25 μg/mL) LZD (0.25–0.5 μg/mL)	Three-dimensional checkerboard	LZD and LFX	+	−	+	−	low
Zhao, 2016 [26]	*M. tuberculosis* H37Rv, 3 MDR-TB clinical isolate	3	+, +, +	Culture	LZD (0.06 to 1 mg/mL) and MFX, LFX, PAS, KAN, CAP, AMK, and CFZ (0.125 mg/Land 8 mg/L).	Checkerboard ^2^	CAP, AMK KAN, LFX, MFX PAS and CFZ	+	−	+	−	High
Li 2019 [27]	*M. tuberculosis* H37Rv, 3 MDR-TB, 2 XDR-TB, 3 Pan- susceptible clinical isolate, and 12 resistant strains to other drugs	30	+, +, +	Culture	CFZ (0.016–2 μg/mL), CAP (0.25–4 μg/mL), MFX (0.016–1 μg/mL).	Checkerboard	CFZ and MFX or CAP	+	−	+	−	Low
Bax 2017 [28]	*M. tuberculosis* Beijing VN 2002-1585 (BE 1585), R-TB	2	+, +, +	Culture	INH (0.125 mg/L), RIF(0.25 mg/L), STR (2 mg/L), EMB (5 mg/L), PAS (0.125 mg/L).	Time-kill kinetics assay	STR, INH, RIF, EMB, PAS and PZA	+	−	−	+	High
Rey-Jurado, 2012 [29]	12 H mono-res or H/S –res, 11 DS clinical isolates	32	+, +, +	Culture	EMB (0.31–5 mg/mL), RIF (0.125–2 mg/mL), OFX (0.125–2 mg/mL) INH (0.025–102.4 mg/mL)	Two-dimensional checkerboard	INH/RIF, and EMB/OFX, RIF and EMB	+	−	+	−	Low
Louie, 2018 [30]	*M. tuberculosis* strain H37Rv and strain 18 b	2	Mutational frequency determination, MIC	Culture	N/A	HFIM	MFX activity Acid, NRP phases	+	+	−	−	High
Cokol, 2017 [31]	Panthotenate and leucine auxothrophic strain of *M. tuberculosis*	Not specified	+, +, +	Culture	N/A	Three-dimensional checkerboard DiaMOND	BDQ + CFZ+ RIF and BDQ + Pa + RIF and BDQ + CFZ+ INH + RIF and CFZ + INH + Pa+ RIF	+	−	+	−	High
Cokol, 2019 [32]	*M. tuberculosis* strain	Not specified	+, +, +	-	N/A	R/ED checkerboard	Pa + ETO and BDQ + CFZ	+	−	+ ^3^	−	High
(Ma, 2019 [33])	Genetic wild-type strain, H37Rv and the TFI strain	14	+, +, +	Culture	N/A	INDIGO-MTB checkerboard assays and high-throughput DiaMOND method	BDQ/ CFZ alone or in a three-drug combination with PZA, EMB, RIF, or INH. INH-RIF-STR	+	+	+	−	High
(Peterson, 2016 [34]).	MTB wild-type H37Rv, ΔRv0324 and ΔRv0880 strains	Not specified	+, −, +	Culture	N/A	INDIGO model, EGRIN and PROM computational models	BDQ and Pa	+	−	+	−	Low

^1^ Mutations in the katG and inhA genes were previously characterised.

^2^ In vitro results were validated in the murine model.

^3^ Fractional Lytic Concentration.

**Table 2:** Summary of drug combinations, synergism and antagonism and models used for its evaluation.

Drug Combination	Synergism/Additive	Antagonism
Computational model INDIGO-MTB, checkerboard assays, and the high-throughput DiaMOND method (Ma, 2019 [33])	BDQ/CFZ alone or in a three-drug combination with PZA, EMB, RIF, or INH. INH-RIF-STR. When Rv1353c is induced, BDQ-STR and CAP-STR shift toward synergy	INH-STR and INH-RIF RIF-MFX. BDQ-STR and CAP-STM shift toward antagonism
BDQ and Pa, INDIGO model, EGRIN, and PROM computational models (Peterson, 2016 [34])	BDQ and Pa Un-induced overexpression of Rv0880 (additive to moderately synergistic BDQ and Pa) Downregulation of the expression of Rv0324 and Rv0880 (considerable synergism)	Induced overexpression of Rv0880 (BDQ and Pa) Increased expression of Rv0324 (BDQ and Pa)
INH and EMB, DNA footprinting, and isothermal titration calorimetry and surface plasmon resonance assays (Zhu, 2018 [37])	INH and EMB	N/A
LZD and RIF, modified checkerboard-REDCA model (Maltempe, 2017 [18])	LZD and RIF (*M. tuberculosis* H37Rv) and 8 (20.5%) clinical isolates. Out of eight, three DS, two INH mono-resistant, and three MDR isolates.	N/A
LZD and RIF (Drusano, 2014 [19])	LZD and RIF interact in a non-significant tendency towards antagonism for killing the wild-type (WT) population.	N/A
INH or EMB interaction with LFX, modified checkerboard assay, REDCA (Calefi-Ferraciol, 2013 [20])	*M. tuberculosis* H37Rv and resistant isolates (EMB and LFX)	INH vs. LFX no synergism
CFZ/Pa/LFX and CFX/Pa/MFX and CFZ/Pa/Ub-8902 Checkerboard assay (López-Gavín, 2015 [21])	CFZ/Pa/LFX, CFZ/Pa/MFX, and CFZ/Pa/Ub-8902 combination (MDR and drug-susceptible isolates)	N/A
MFX/Pa interaction in Log, Acid and NRP phases using a 9 by 8 well checkerboard assay (Miranda Silva, 2019 [22])	MFX and Pa additive for all metabolic state	N/A
LZD/BDQ in Log, Acid, and NRP Phases,9 by 8 well Checkerboard assay (Miranda Silva, 2018 [23]),	LZD and BDQ is additive for bacterial killing in both strains for all metabolic states.	N/A
BDQ/MFX/GFX/CFZ, and LZD, checkerboard assay (Pang, 2019 [24])	BDQ combination with MFX, GFX, CFZ, and LZD for treatment XDR-TB	XDR-TB isolates for BDQ-MFX, BDQ-GFX, BDQ-LZD, and BDQ-CFZ
LZD and LFX three-dimensional checkerboard (Santos, 2018 [25])	40% of resistant clinical isolates INH/RIF/LFX and 50% resistant clinical isolates INH/RIF/LZD, with a better synergism observed for INH and RIF combined to LVX or LZD at 1/4 MIC	N/A
LZD and CAP, AMK KAM, LFX, MFX, PAS, and CFZ, checkerboard assay (Zhao, 2016 [26])	LZD/CAP/ LZD/PAS, LZD/LFX and LZD/AMK showed partial synergism in 3/4, 2/4, 1/4 isolates, respectively (REDCA)	N/A
CFZ with MFX or CAP checkerboard assay (Li, 2019 [27])	CFZ/CAP CFZ/MFX.	M/XDR strains in increased concentration of CFZ in CFZ/CAP and CFZ/MFX combination
STR, INH, RIF, EMB, Pas and PZA time-kill kinetics (Bax, 2017 [28])	INH/RIF at clinically used concentrations	N/A
INH/RIF, EMB/OFX RIF/EMB, two-dimensional checkerboard assay (Rey Jurado, 2012 [29])	INH, RIF and EMB synergism in the INH drug res isolates OFX, RIF and EMB in the res and DS isolates	N/A
High-throughput combinational screening, checkerboard and DiAMOND (Cokol, 2017 [31])	BDQ + CFZ + INH, BDQ + CFZ + RIF and BDQ + Pa + RIF and four-way combinations BDQ + CFZ + INH + RIF and CFZ + INH+ Pa+ RIF	N/A
Pa + ETO and BDQ + CFZ, R/ED checkerboard assay (Cokol, 2019 [32])	Pa + ETO and BDQ + CFZ is against RIF-resistant *M. tuberculosis*. Pa + VAN and FUS + CFZ CFZ + FUS and (LAS) + Pa against MDR isolates CFZ + INH and ETO + RIF	N/A

## Supplementary Materials

The following is available online at https://www.mdpi.com/article/10.3390/microorganisms10030514/s1, Table S1: Summary of the drug targets, products and mechanism of action. References [54,55,56,57,58,59,60,61,62,63,64,65,66,67,68,69,70,71,72,73,74,75,76,77,78,79,80,81,82,83] are cited in the supplementary materials. Table S2: Abbreviation.
