# Supplementary material for: Pre-Clinical Tools for Predicting Drug Efficacy in Treatment of Tuberculosis
Source: Microorganisms. 2022 Feb 26;10(3):514. doi: 10.3390/microorganisms10030514 (PMC8956012; doi:10.3390/microorganisms10030514)
Supplement: Supplementary file 1 [file microorganisms-10-00514-s001.zip › microorganisms-1596562-Table S1.pdf]

Table S 1 Summary of the drug targets, products and mechanism of action.

Drug targets	Product	Functional activity	Mechanism of action
**Ddn**<br><br>**fgd1**	Encodes deazaflavin-dependent nitro-reductase<br><br>encodes F420-dependent glucose-6-phosphate dehydrogenase	Mycolic acid biosynthesis.	(Pa) Inhibits the cell wall synthesis [1,2]
**rplC (Rv0701)**<br><br>**rrl (MTB000020)**	Encodes 50S ribosomal L3 protein<br><br>Ribosomal RNA 23 S	Ribosome peptidyl transferase center formation<br><br>Stable RNAs	(LZD) Inhibits protein synthesis [3-5]
**MmpL5 (RV0678)**<br><br>**(Rv1979c)**	Conserved protein<br><br>conserved permease	Transcription repressor for efflux pump<br><br>Transportation of amino acid across membrane	(CFZ) Inhibits mycobacterial growth and binds to mycobacterial DNA [6,7]
**Rv0678**<br><br>**atpE Rv1305**	Conserved protein<br><br>the subunit C of the ATP synthase	Regulation of multi-substrate efflux pump<br><br>disturbs inhibition against bacterial ATP synthase C	(BDQ) Inhibition of mycobacterial ATP synthetase [8-10]
**katG,(1908c)**<br><br>**inhA (Rv 1484)**<br><br>**kasA (Rv2245)**	Catalase peroxidase<br><br>NADH dependant enoyl ACP reductase<br><br>β-Ketoacyl acyl carrier protein synthase	Intracellular survival of mycobacteria<br><br>Mycolic acid biosynthesis<br><br>Fatty acid biosynthesis	(INH) Inhibition of cell wall mycolic acid synthesis [11-13]
**rpoB (Rv 0667)**<br><br>**rpoA (Rv3457c)**	β-subunit of RNA polymerase<br><br>α-Subunit of RNA polymerase	Catalysis DNA transcription into RNA	(RIF) Inhibition of RNA synthesis [14,15]
**embA Rv3794**<br><br>**embB (Rv3795)**<br><br>**emb C (Rv3793)**<br><br>**EtbR (Rv0273c)**	Probable arabinosyl transferase A<br><br>Probable arabinosyl transferase B<br><br>Probable arabinosyltransferase C<br><br>Transcriptional regulator in M. tuberculosis	Biosynthesis of mycobacterial cell wall	(EMB) Inhibition of cell wall arabinogalactan biosynthesis [16,17]
**pncA (Rv2043c)**<br><br>**RpsA (Rv 1630)**	Pyrazinamidase/nicotinamidase)<br><br>30 S ribosomal protein S1	Converts the pro-drug Z into pyrazinoic acid (POA)<br><br>mRNA translation with a shine dalgarno purine rich sequence	(PZA) Reduction of membrane energy; inhibition of trans-translation; inhibition of pantothenate and coenzyme A synthesis [18,19]

**rpsL (Rv0682)**<br><br>**rrs ((MTB000019)**	30 S ribosomal protein 12Rpsl<br><br>16 S ribosomal RNA	Initiation of translation<br><br>Stable RNAs	(STR) Inhibition of protein synthesis [20,21]
**gyrA (Rv006)**<br><br>**gyrB (0005)**	DNA gyrase subunit A<br><br>DNA gyrase subunit B	Negatively supercoils closed circular double-standard DNA	Fluoroquinalone Inhibition of DNA synthesis [22,23]
**rrs, (MTB00019)**<br><br>**eis, (Rv2416)**<br><br>**tlyA (Rv1694)**	16 ribosomal RNA<br><br>Aminoglycoside N-acetyl transferase<br><br>2'-O- methyltransferase	Stable RNAs<br><br>Acetylation intracellular survival<br><br>Methylates 16S and 23 S rRNAs	(CAP, AMK, KAM) Inhibition of protein synthesis [24-26]
**hyA (Rv 2764c)**<br><br>**folC (Rv2447c),**<br><br>**ribD (Rv2671)**	Thymidylate synthase<br><br>Folylpolyglutamate synthase protein<br><br>Bifunctional enzyme riboflavin biosynthesis protein	Deoxiribonucleotide biosynthesis<br><br>Conversion of folates to polyglutamate derivates<br><br>Riboflavin biosynthesis	(PAS) Inhibition of folic acid and thymine nucleotide metabolism [27-29]
**ethA (Rv3854c)**<br><br>**inhA (Rv1484)**<br><br>**KasA (Rv 2245)**	Monooxygenase EthA<br><br>NADH dependant enoyl ACP reductase<br><br>β-ketoacyl acyl carrier protein synthase	Activation of pro-drug Eth<br><br>Mycolic acid biosynthesis<br><br>Involved in fatty acid biosynthesis	(ETO) Inhibition of cell wall mycolic acid synthesis [30-32]

1. Haver, H.L.; Chua, A.; Ghode, P.; Lakshminarayana, S.B.; Singhal, A.; Mathema, B.; Wintjens, R.; Bifani, P. Mutations in genes for the F420 biosynthetic pathway and a nitroreductase enzyme are the primary resistance determinants in spontaneous in vitro-selected PA-824-resistant mutants of Mycobacterium tuberculosis. *Antimicrob Agents Chemother* **2015**, *59*, 5316-5323, doi:10.1128/AAC.00308-15.
2. Manjunatha, U.; Boshoff, H.I.; Barry, C.E. The mechanism of action of PA-824: Novel insights from transcriptional profiling. *Commun Integr Biol* **2009**, *2*, 215-218, doi:10.4161/cib.2.3.7926.
3. Swaney, S.M.; Aoki, H.; Ganoza, M.C.; Shinabarger, D.L. The oxazolidinone linezolid inhibits initiation of protein synthesis in bacteria. *Antimicrob Agents Chemother* **1998**, *42*, 3251-3255, doi:10.1128/AAC.42.12.3251.
4. Beckert, P.; Hillemann, D.; Kohl, T.A.; Kalinowski, J.; Richter, E.; Niemann, S.; Feuerriegel, S. rplC T460C identified as a dominant mutation in linezolid-resistant Mycobacterium tuberculosis strains. *Antimicrobial agents and chemotherapy* **2012**, *56*, 2743-2745.
5. Balasubramanian, V.; Solapure, S.; Iyer, H.; Ghosh, A.; Sharma, S.; Kaur, P.; Deepthi, R.; Subbulakshmi, V.; Ramya, V.; Ramachandran, V.; et al. Bactericidal activity and mechanism of action of AZD5847, a novel oxazolidinone for treatment of tuberculosis. *Antimicrob Agents Chemother* **2014**, *58*, 495-502, doi:10.1128/AAC.01903-13.
6. Cholo, M.C.; Mothiba, M.T.; Fourie, B.; Anderson, R. Mechanisms of action and therapeutic efficacies of the lipophilic antimycobacterial agents clofazimine and bedaquiline. *J Antimicrob Chemother* **2017**, *72*, 338-353, doi:10.1093/jac/dkw426.
7. Hartkoorn, R.C.; Uplekar, S.; Cole, S.T. Cross-resistance between clofazimine and bedaquiline through upregulation of MmpL5 in Mycobacterium tuberculosis. *Antimicrobial agents and chemotherapy* **2014**, *58*, 2979-2981.

8. Andries, K.; Verhasselt, P.; Guillemont, J.; Gohlmann, H.W.; Neefs, J.M.; Winkler, H.; Van Gestel, J.; Timmerman, P.; Zhu, M.; Lee, E.; et al. A diarylquinoline drug active on the ATP synthase of Mycobacterium tuberculosis. *Science* **2005**, *307*, 223-227, doi:10.1126/science.1106753.
9. Koul, A.; Dendouga, N.; Vergauwen, K.; Molenberghs, B.; Vranckx, L.; Willebrords, R.; Ristic, Z.; Lill, H.; Dorange, I.; Guillemont, J.; et al. Diarylquinolines target subunit c of mycobacterial ATP synthase. *Nat Chem Biol* **2007**, *3*, 323-324, doi:10.1038/nchembio884.
10. Huitric, E.; Verhasselt, P.; Koul, A.; Andries, K.; Hoffner, S.; Andersson, D.I. Rates and mechanisms of resistance development in Mycobacterium tuberculosis to a novel diarylquinoline ATP synthase inhibitor. *Antimicrob Agents Chemother* **2010**, *54*, 1022-1028, doi:10.1128/AAC.01611-09.
11. Ramaswamy, S.V.; Reich, R.; Dou, S.J.; Jasperse, L.; Pan, X.; Wanger, A.; Quitugua, T.; Graviss, E.A. Single nucleotide polymorphisms in genes associated with isoniazid resistance in Mycobacterium tuberculosis. *Antimicrob Agents Chemother* **2003**, *47*, 1241-1250, doi:10.1128/AAC.47.4.1241-1250.2003.
12. Vilcheze, C.; Wang, F.; Arai, M.; Hazbon, M.H.; Colangeli, R.; Kremer, L.; Weisbrod, T.R.; Alland, D.; Sacchettini, J.C.; Jacobs, W.R., Jr. Transfer of a point mutation in Mycobacterium tuberculosis inhA resolves the target of isoniazid. *Nat Med* **2006**, *12*, 1027-1029, doi:10.1038/nm1466.
13. Massengo-Tiasse, R.P.; Cronan, J.E. Diversity in enoyl-acyl carrier protein reductases. *Cell Mol Life Sci* **2009**, *66*, 1507-1517, doi:10.1007/s00018-009-8704-7.
14. Thirumurugan, R.; Kathirvel, M.; Vallayyachari, K.; Surendar, K.; Samrot, A.V.; Muthaiah, M. Molecular analysis of rpoB gene mutations in rifampicin resistant Mycobacterium tuberculosis isolates by multiple allele specific polymerase chain reaction in Puducherry, South India. *J Infect Public Health* **2015**, *8*, 619-625, doi:10.1016/j.jiph.2015.05.003.
15. Zhang, Q.; An, X.; Liu, H.; Wang, S.; Xiao, T.; Liu, H. Uncovering the resistance mechanism of Mycobacterium tuberculosis to rifampicin due to RNA polymerase H451D/Y/R mutations from computational perspective. *Frontiers in chemistry* **2019**, *7*, 819.
16. Safi, H.; Lingaraju, S.; Amin, A.; Kim, S.; Jones, M.; Holmes, M.; McNeil, M.; Peterson, S.N.; Chatterjee, D.; Fleischmann, R.; et al. Evolution of high-level ethambutol-resistant tuberculosis through interacting mutations in decaprenylphosphoryl-beta-D-arabinose biosynthetic and utilization pathway genes. *Nat Genet* **2013**, *45*, 1190-1197, doi:10.1038/ng.2743.
17. Zhu, C.; Liu, Y.; Hu, L.; Yang, M.; He, Z.G. Molecular mechanism of the synergistic activity of ethambutol and isoniazid against Mycobacterium tuberculosis. *J Biol Chem* **2018**, *293*, 16741-16750, doi:10.1074/jbc.RA118.002693.
18. Jureen, P.; Werngren, J.; Toro, J.C.; Hoffner, S. Pyrazinamide resistance and pncA gene mutations in Mycobacterium tuberculosis. *Antimicrob Agents Chemother* **2008**, *52*, 1852-1854, doi:10.1128/AAC.00110-08.
19. Njire, M.; Tan, Y.; Mugweru, J.; Wang, C.; Guo, J.; Yew, W.; Tan, S.; Zhang, T. Pyrazinamide resistance in Mycobacterium tuberculosis: Review and update. *Adv Med Sci* **2016**, *61*, 63-71, doi:10.1016/j.advms.2015.09.007.
20. Sharma, D.; Cukras, A.R.; Rogers, E.J.; Southworth, D.R.; Green, R. Mutational analysis of S12 protein and implications for the accuracy of decoding by the ribosome. *J Mol Biol* **2007**, *374*, 1065-1076, doi:10.1016/j.jmb.2007.10.003.
21. Springer, B.; Kidan, Y.G.; Prammananan, T.; Ellrott, K.; Bottger, E.C.; Sander, P. Mechanisms of streptomycin resistance: selection of mutations in the 16S rRNA gene conferring resistance. *Antimicrob Agents Chemother* **2001**, *45*, 2877-2884, doi:10.1128/AAC.45.10.2877-2884.2001.
22. Nosova, E.Y.; Bukatina, A.A.; Isaeva, Y.D.; Makarova, M.V.; Galkina, K.Y.; Moroz, A.M. Analysis of mutations in the gyrA and gyrB genes and their association with the resistance of Mycobacterium tuberculosis to levofloxacin, moxifloxacin and gatifloxacin. *J Med Microbiol* **2013**, *62*, 108-113, doi:10.1099/jmm.0.046821-0.
23. Aubry, A.; Pan, X.S.; Fisher, L.M.; Jarlier, V.; Cambau, E. Mycobacterium tuberculosis DNA gyrase: interaction with quinolones and correlation with antimycobacterial drug activity. *Antimicrob Agents Chemother* **2004**, *48*, 1281-1288, doi:10.1128/AAC.48.4.1281-1288.2004.
24. Reeves, A.Z.; Campbell, P.J.; Sultana, R.; Malik, S.; Murray, M.; Plikaytis, B.B.; Shinnick, T.M.; Posey, J.E. Aminoglycoside cross-resistance in Mycobacterium tuberculosis due to mutations in the 5' untranslated region of whiB7. *Antimicrob Agents Chemother* **2013**, *57*, 1857-1865, doi:10.1128/AAC.02191-12.
25. Sowajassatakul, A.; Prammananan, T.; Chaiprasert, A.; Phunpruch, S. Molecular characterization of amikacin, kanamycin and capreomycin resistance in M/XDR-TB strains isolated in Thailand. *BMC Microbiol* **2014**, *14*, 165, doi:10.1186/1471-2180-14-165.

26. Smith, T.; Wolff, K.A.; Nguyen, L. Molecular biology of drug resistance in Mycobacterium tuberculosis. *Pathogenesis of Mycobacterium tuberculosis and its Interaction with the Host Organism* **2012**, 53-80.
27. Zhao, F.; Wang, X.D.; Erber, L.N.; Luo, M.; Guo, A.Z.; Yang, S.S.; Gu, J.; Turman, B.J.; Gao, Y.R.; Li, D.F.; et al. Binding pocket alterations in dihydrofolate synthase confer resistance to para-aminosalicylic acid in clinical isolates of Mycobacterium tuberculosis. *Antimicrob Agents Chemother* **2014**, *58*, 1479-1487, doi:10.1128/AAC.01775-13.
28. Rengarajan, J.; Sassetti, C.M.; Naroditskaya, V.; Sloutsky, A.; Bloom, B.R.; Rubin, E.J. The folate pathway is a target for resistance to the drug para-aminosalicylic acid (PAS) in mycobacteria. *Mol Microbiol* **2004**, *53*, 275-282, doi:10.1111/j.1365-2958.2004.04120.x.
29. Mathys, V.; Wintjens, R.; Lefevre, P.; Bertout, J.; Singhal, A.; Kiass, M.; Kurepina, N.; Wang, X.M.; Mathema, B.; Baulard, A.; et al. Molecular genetics of para-aminosalicylic acid resistance in clinical isolates and spontaneous mutants of Mycobacterium tuberculosis. *Antimicrob Agents Chemother* **2009**, *53*, 2100-2109, doi:10.1128/AAC.01197-08.
30. Grant, S.S.; Wellington, S.; Kawate, T.; Desjardins, C.A.; Silvis, M.R.; Wivagg, C.; Thompson, M.; Gordon, K.; Kazyanskaya, E.; Nietupski, R.; et al. Baeyer-Villiger Monooxygenases EthA and MymA Are Required for Activation of Replicating and Non-replicating Mycobacterium tuberculosis Inhibitors. *Cell Chem Biol* **2016**, *23*, 666-677, doi:10.1016/j.chembiol.2016.05.011.
31. Carette, X.; Blondiaux, N.; Willery, E.; Hoos, S.; Lecat-Guillet, N.; Lens, Z.; Wohlkonig, A.; Wintjens, R.; Soror, S.H.; Frenois, F.; et al. Structural activation of the transcriptional repressor EthR from Mycobacterium tuberculosis by single amino acid change mimicking natural and synthetic ligands. *Nucleic Acids Res* **2012**, *40*, 3018-3030, doi:10.1093/nar/gkr1113.
32. Rueda, J.; Realpe, T.; Mejia, G.I.; Zapata, E.; Rozo, J.C.; Ferro, B.E.; Robledo, J. Genotypic analysis of genes associated with independent resistance and cross-resistance to isoniazid and ethionamide in Mycobacterium tuberculosis clinical isolates. *Antimicrobial agents and chemotherapy* **2015**, *59*, 7805-7810.
