# Supplementary material for: Pre-Clinical Tools for Predicting Drug Efficacy in Treatment of Tuberculosis
Source: Microorganisms. 2022 Feb 26;10(3):514. doi: 10.3390/microorganisms10030514 (PMC8956012; doi:10.3390/microorganisms10030514)
Supplement: Supplementary file 1 [file microorganisms-10-00514-s001.zip › microorganisms-1596562-Table S2.pdf]

Table S 2 Abbreviation

AMK	Amikacin
AUC	Area under the curve
BDQ	Bedaquiline
CM	Capreomycin
CFU	Colony Forming Unit
CFZ	Clofazimine
DNA	Deoxyribonucleic acid
DS-TB	Drug sensitive TB
EGRIN	Environment and Gene Regulatory Influence Network
EMB	Ethambutol
EBA	Early bactericidal activity
ETH	Ethionamide
FDA	Food and Drug Authority
FICI	Fractional inhibitory concentration index
FUS	Fusidic Acid
GFX	Gatifloxacin
HFIM	Hollow Fiber infection model
HIV	Human Immunodeficiency Virus
INH	Isoniazid
KM	Kanamycin
LAS	Lassomycin

LFX	Levofloxacin
LZD	Linezolid
MIC	Minimum inhibitory concentration
MFX	Moxifloxacin
MDR-TB	Multi drug resistant TB
NRP	Non replicating phase
PAS	Para-amino-salicylate-Sodium
PA	Pretomanid
PRISMA	Preferred Reporting Items for Systematic Reviews and Meta-Analyses
PROM	Probabilistic Regulation of Metabolism
PZA	Pyrazinamid
REDCA	Resazurin drugs concentrations assays
RIF	Rifampicin
SLD	Second-line anti-TB drugs
STR	Streptomycin
TRN	Transcriptional Regulatory Network
URSA	Universal Response Surface Approach
VAN	Vancomycin
WHO	World health Organization
XDR-TB	Extensively drug resistant TB
